# Polymorphic Microsatellite Loci Isolated from the *Squalidus argentatus* Using PCR-Based Isolation of Microsatellite Arrays (PIMA)

**DOI:** 10.3390/ijms12095666

**Published:** 2011-09-02

**Authors:** Yang Sun, Hung-Du Lin, Wen-Qiao Tang, Yu-Min Ju, Zhi-Zhi Liu, Dong Liu, Jin-Quan Yang

**Affiliations:** 1 Laboratory of Fishes, Shanghai Ocean University, Shanghai, 201306, China; E-Mails: sunyang_zhuiyi@163.com (Y.S.); wqtang@shou.edu.cn (W.-Q.T.); zzliu@shou.edu.cn (Z.-Z.L); dliu@shou.edu.cn (D.L.); 2 The Affiliated School of National Tainan First Senior High School, Tainan 701, Taiwan; E-Mail: varicorhinus@hotmail.com; 3 National Museum of Marine Biology and Aquarium, Pingtung 900, Taiwan; E-Mail: yumine@nmmba.gov.tw

**Keywords:** microsatellite, conservation, PIMA, RAPD-PCR enrichment, *Squalidus argentatus*

## Abstract

*Squalidus argentatus* (Sauvage and Dabry de Thiersant 1874) is a small-sized freshwater fish which is distributed in Mainland China, Hainan Island and Taiwan. The populations of *S. argentatus* have dropped sharply probably due to overharvesting and water pollution recently. Eleven polymorphic microsatellite markers were developed for the cyprinid fish *S. argentatus*. These new markers were tested on 43 individuals collected from Yangtze River and Qiantang River. The number of alleles, observed and expected heterozygosity per locus, in two populations ranged from 3 to 14, from 0.333 to 0.954 and from 0.480 to 0.928, respectively. Only two loci are significantly deviated from Hardy–Weinberg expectations due to the heterozygote deficiency. No significant linkage disequilibrium was detected between the pairwise comparisons of these loci. These polymorphic microsatellite loci will enable us to study the genetic variation, population structure, and conservation genetics of this species in the future.

## 1. Introduction

Genus *Squalidus argentatus* (Sauvage and Dabry de Thiersant 1874) (Cypriniformes, Cyprinidae) consists of a group of small-sized freshwater fishes widely found in the lower or middle reaches of Eastern Asia, including Mainland China, Hainan Island and Taiwan. There are at least seven valid species of *Squalidus* found in mainland China and two endemic species reported from Taiwan [1]. Among them, *S. argentatus* is the only species commonly found in above mentioned areas [2]. Within the last decade, the wild resource of *S. argentatus* has experienced a dramatic decrease probably owing to over-fishing and environmental destruction. In order to develop effective strategies for fishery management and conservation, information, such as the population genetic structure and genetic diversity and divergence of *S. argentatus*, is important. DNA-based genetic analyses can yield valuable information for assessing levels of variation and population genetic structure in freshwater fishes. Because of their high level of polymorphism and co-dominant inheritance in Mendelian fashion, microsatellites have been widely used as DNA markers in the studies of population structure and conservation genetics of freshwater fishes [3]. Although many studies related to taxonomy [1], embryonic development [4] and phylogenetic relationship [5] of *S. argentatus*, have been conducted, there is no report available on molecular markers isolation for this species. The microsatellite markers identified will allow the undertaking of further genetic studies on *S. argentatus* and provide valuable genetic information to assist the management and culture of this resource.

## 2. Experimental Section

### 2.1. Isolation of Microsatellite Markers

In the present study, we developed 11 polymorphic microsatellite loci isolated using PCR-based isolation of microsatellite arrays (PIMA) of *Squalidus argentatus*. In brief, total genomic DNA was extracted from muscle tissue or fins preserved in 95% ethanol, by proteinase K digestion at 55 °C. DNA was purified by traditional phenol-chloroform protocol and ethanol precipitation [6]. The PCR library was obtained by the PCR isolation of microsatellite arrays (PIMA) method that is based on random amplified polymorphic DNA-polymerase chain reaction (RAPD-PCR) enrichment [7–9]. It takes advantage of the fact that the RAPD fragments contain microsatellite repeats more frequently than random genomic clones [10]. Amplification of 20–100 ng of DNA was performer in a 15 μL final volume with 0.2 mM of each dNTP, 2 mM MgCl_2_, 0.5 U Taq polymerase (Promega), and 5 pmols of one RAPD primer. Several RAPD primers were used to amplify fragments from the target species’ genome in separate reactions. Reactions of PCR amplification were conducted in a thermal cycler (Eppendorf Mastercycler) using the following conditions: initial denaturing at 94 °C for 3 min; 40 cycles of 94 °C denaturing for 50 s, 37 °C annealing for 1 min, 72 °C extension for 1 min; and 72 °C for 10 min. RAPD-PCR products were size-selected to preferentially obtain small fragments (500–1200 bp). Approximately 100 ng of PCR product was ligated into a pMD 19-T vector (Takara) according to the manufacturer’s instructions, and the ligation mixture was transformed into *Escherichia coli* DH5α competent cells. Clones were screened of the library for microsatellites by PCR with both vector primers plus a microsatellite specific primer (GT and GA repeats) [8].

In positive clones, the repeat-specific and vector primers amplified DNA fragments that contain microsatellites, whereas no amplification was found in negative clones. Plasmid DNA from positives was purified using the High-Speed Plasmid Mini Kit (Geneaid). Both strands of the DNA insert were sequenced. Sequencing of positive insert PCRs was conducted by Sangon Biotec (Shanghai) Co., Ltd. with an ABI PRISM 3730 sequencer using the BigDye Terminator kit (Applied Biosystems) and by using vector primers. Primer pairs were newly designed for eleven microsatellite sequences with the program PRIMER 3 software [11]. Preliminary assessment of polymorphism was performed on a few individuals. Reactions were performed in a total volume of 15 μL containing 10 ng of genomic DNA, 0.2 mM dNTP, 2 mM MgCl_2_, and 0.12 μM of each primer. PCR reactions were as follows: 94 °C for 4 min followed by 40 cycles at 94 °C for 30 s, 30 s–50 s at primer-specific annealing temperature (Table 1), 72 °C for 45 s and a final extension step at 72 °C for 10 min. PCR product was analyzed automatically by the QIAxcel system in combination with QIAxcel DNA High Resolution Kit (QIAGEN, German). The method was selected OM700. PBR322/MSP I marker (TIANGEN, China) was used as standard for scoring.

### 2.2. Data Analysis

Allele frequency, observed (*H*_O_) and expected (*H*_E_) heterozygosities were calculated. All loci were tested for fitness to the Hardy–Weinberg equilibrium (HWE), and all pairwise combinations of loci were tested for linkage disequilibrium. All these parameters and tests were computed using ARLEQUIN 3.5 [12]. Results of the tests were corrected for multiple comparisons by applying sequential Bonferroni corrections [13]. The software MICROCHECKER was employed to infer the most probable technical cause of HWE departures, including null alleles, allelic dropout due to short allele dominance, and errors made during the scoring of alleles with ‘stutter’ on our data [14].

## 3. Results and Discussion

A total of 43 *Squalidus argentatus*, including 21 from Yangtze River and 22 from Qiantang River, were collected and their levels of genetic diversity were estimated by 11 microsatellite loci. Locus designation, GenBank accession number, repeat motif, PCR product size range and number of alleles for the 11 microsatellite markers are listed in Table 1. The number of alleles on each locus ranged from 3 to 11 (average 6.364) in Yangtze River and from 4 to 14 (average 8.636) in Qiantang River. As shown in Table 2, the *H*_E_ and *H*_O_ ranged from 0.480–0.923 (average of 0.767) and 0.333–0.857 (average of 0.668), respectively, in Yangtze River. The *H*_E_ and *H*_O_ ranged from 0.717–0.928 (average of 0.830) and 0.476–0.954 (average of 0.747), respectively, in Qiantang River. One locus in Yangtze River (MISA10) and in Qiantang River (MISA11) is significantly deviated from Hardy–Weinberg equilibrium after Bonferroni’s correction (*P* < 0.001) due to the heterozygote deficiency, respectively (Table 2). These deviations may have resulted from null alleles or small population size associated with human disturbances and habitat loss [15]. With MICRO-CHECKER utility, null alleles were found in MISA11 locus (*P* < 0.05), but for stuttering and allelic dropout tests, no evidences were found in all loci (*P* > 0.05). After applying sequential Bonferroni corrections to compensate for multiple statistical tests, no evidence of genotypic linkage disequilibrium at any pair of loci was found. Microsatellite makers described here should be useful to monitor population dynamics and to determine dispersal patterns and genetic diversity within and between populations of this species. These polymorphic microsatellite loci will be used to investigate population structure and gene flow among *S. argentatus* populations and are expected to provide a better understanding about the ecology of the species, and consequently a basis for a sustainable management framework in the future.

## 4. Conclusions

The wild resource of *Squalidus argentatus* has experienced a dramatic decrease in the last decade. Therefore, there is an urgent need for the development and application of microsatellites to provide an effective tool for investigation of genetic variation and population structure in *S. argentatus*. In conclusion, we report, these 11 polymorphic microsatellite loci presented in this study were the first set of microsatellite markers designed specifically for *S. argentatus*, and provides the ground work for further studies on the genetic structure, gene flow, conservation management and molecular evolution of this vulnerable species.

## Figures and Tables

**Table 1:** The forward (F) and reverse (R) primer sequences, repeat motif, size range and Tm, annealing temperature for eleven microsatellite loci of *Squalidus argentatus*.

Locus	Genbank Accession No.	Primer sequence(5′ to 3′)	Repeat motif	Size range (bp)	Tm °C
MISA01	JN582002	F: TCTGACCCAACGGTTTCTGC	(TG)_10_	222–252	63
		R: GGACACCTGCTGACGCTCTT			
MISA02	JN582003	F: AGCTAACTGAGCTGCATACAAC	(AG)_18_	262–280	59
		R: GGTGGTGGAACATAAAGTGACA			
MISA03	JN582004	F: AGCCAACCTGCGTCGTTATCTAC	(TG)_14_	186–222	63
		R: GGGACTCACAACCAGTTTTGGTT			
MISA04	JN582005	F: ATCAAAGGTAAGAGAACATCAGCG	(CT)_21_	212–308	61
		R: TCAATTAAATCCTTCCCGGTGTAC			
MISA05	JN582006	F: TTGAGCATGACAGAGCACACAGAATC	(AG)_8_G(GA)_27_	298–370	63
		R: CGAAACCAGTGAATGCCAAAGTCTC			
MISA06	JN582007	F: TGGCTCCTTTCACACCCGT	(CAT)_6…_(TC)_10…_(TC)_6…_(TG)_6_	202–280	63
		R: AGGCAGGAGAGGAAGAGCG			
MISA07	JN582008	F: AGGACACCTGCTGACGCTCTT	(AC)_9_	224–246	65
		R: CTCTGACCCAACGGTTTCTGC			
MISA08	JN582009	F: TGACACAGTGTAGACTTTCCAAAC	(TC)_15_(TG)_9_	202–278	61
		R: GAACTCAGCAGAAATAGAAACCAT			
MISA09	JN582010	F: ATCGCTTCAGACTCACCTCATC	(TG)_31_	326–380	63
		R: AAACCTCAAATTCGACCAAAAT			
MISA10	JN582011	F: ACACTGAGGAGTTCAAATAAAGCC	(TG)_5…_(TG)_6_	182–194	61.5
		R: TCATCTTCTAAGAGAGCAGGAGTG			
MISA11	JN582012	F: CGATCATCATGGTGTTTCCTGC	(AC)_9…_(CA)_15_	246–346	61
		R: TCGGGTTGTATTGCTCTTCAGT			

**Table 2:** List of eleven microsatellite loci for *Squalidus argentatus* in Yangtze River and Qiantang River. Number of alleles (*N*_A_), expected (*H*_E_) and observed (*H*o) heterozgosities and significance of deviation from Hardy–Weinberg equilibrium for microsatellite loci.

	Yangtze River				Qiantang River			
	*N*_A_	*H*o	*H*_E_	HWE *P*-value	*N*_A_	*H*o	*H*_E_	HWE *P*-value
MISA01	6	0.73333	0.81839	0.11512	6	0.68182	0.80655	0.05309
MISA02	4	0.64706	0.75758	0.23241	4	0.95455	0.74947	0.12959
MISA03	7	0.81250	0.73185	0.03699	6	0.70588	0.82353	0.32288
MISA04	8	0.85714	0.88153	0.13961	14	0.81818	0.92812	0.29437
MISA05	11	0.82353	0.91979	0.32180	14	0.90909	0.91966	0.02107
MISA06	11	0.71429	0.92328	0.11708	12	0.72727	0.87844	0.29985
MISA07	4	0.63158	0.66145	0.12610	5	0.81818	0.71776	0.51218
MISA08	7	0.66667	0.84762	0.38511	13	0.77273	0.92600	0.06433
MISA09	5	0.46667	0.69885	0.22965	8	0.81818	0.85095	0.47429
MISA10	4	0.66667	0.71661	0.00038	4	0.54545	0.74841	0.00152
MISA11	3	0.33333	0.48046	0.03974	9	0.47619	0.78513	0.00000
**mean**	6.364	0.66843	0.76704		8.636	0.74796	0.83037	
